# Book Notices

**Published:** 1866-11

**Authors:** 


### EDITORIAL.

### BOOK NOTICES.

*A Guide to the Practical Study of Diseases of the Eye, etc.* By JAMES DIXON, F.R.C.S., etc. (From the Third London Edition.) Philadelphia: Lindsay & Blakiston. 1866. For sale by S. C. Griggs & Co., Chicago.

An elementary work on diseases of the eye, suitable in all respects for the use of students, would be a most valuable addition to the list of excellent text-books in the various departments of medicine.

The little work of Mr. Dixon is certainly one of the best yet published. The student who, in connection with clinical instruction on diseases of the eye, can impress upon his memory all the principles discussed in this work, will be better enabled to study intelligently the more extended works on ophthalmology.

There are, however, many faults in the work. The description of symptoms is often very imperfect. We can scarcely conceive how a practitioner's own experience, or his knowledge of the experience of others, should lead him to almost wholly ignore the use of atropine in the treatment of iritis.

Mr. Dixon states that it is the function of the oblique muscles of the eye to keep the perpendicular diameter of each cornea (meridian of the globe) parallel with that of the other, when the head is inclined from side to side towards the shoulder. We have been taught to believe this theory of the function of the oblique muscles had long since been absolutely overthrown by direct positive proof, and that the true function of these muscles consists in maintaining the meridian of the two globes parallel in the movements of the eyes upwards or downwards to the left or right.

